# Construct validation of a non-exercise measure of cardiorespiratory fitness in older adults

**DOI:** 10.1186/1471-2458-10-59

**Published:** 2010-02-08

**Authors:** Emily L Mailey, Siobhan M White, Thomas R Wójcicki, Amanda N Szabo, Arthur F Kramer, Edward McAuley

**Affiliations:** 1 Department of Kinesiology & Community Health, University of Illinois at Urbana-Champaign, 906 S Goodwin Ave, Urbana, IL 61801, USA; 2 Department of Psychology, University of Illinois at Urbana-Champaign, 603 E. Daniel St, Champaign, IL 61820, USA

## Abstract

**Background:**

Cardiorespiratory fitness (CRF) is associated with a decreased risk of all-cause mortality but is rarely assessed in medical settings due to burdens of time, cost, risk, and resources. The purpose of this study was to test the construct validity of a regression equation developed by Jurca and colleagues (2005) to estimate CRF without exercise testing in community dwelling older adults.

**Methods:**

Participants (n = 172) aged 60 to 80 years with no contraindications to submaximal or maximal exercise testing completed a maximal graded exercise test (GXT) and the submaximal Rockport 1-mile walk test on separate occasions. Data included in the regression equation (age, sex, body mass index, resting heart rate, and physical activity) were obtained via measurement or self-report. Participants also reported presence of cardiovascular conditions.

**Results:**

The multiple R for the regression equation was .72, *p < .001 *and CRF estimated from this equation was significantly correlated with the MET value from the GXT (*r *= 0.66) and with CRF estimated from submaximal field testing (*r *= 0.67). All three CRF indices were significantly and inversely associated with reporting more cardiovascular conditions.

**Conclusions:**

This research provides preliminary evidence that a non-exercise estimate of CRF is at least as valid as field test estimates of CRF and represents a low-risk, low-cost, and expedient method for estimating fitness in older adults.

## Background

Cardiorespiratory fitness (CRF) is associated with a decreased risk of all-cause mortality and chronic illnesses, especially cardiovascular disease [1,2]. Additionally, cross-sectional studies and randomized clinical trials suggest that CRF is associated with brain structure and function [3] and that improvements in CRF brought about by exercise training are implicated in the restoration of neural and cognitive functioning in older adults [4-6]. Whereas the consideration of common risk factors such as physical inactivity and overweight status can provide information relative to the health status of an individual, CRF is a multifaceted construct that reflects the overall efficiency of the cardiovascular system and takes these other common health indicators into account.

Although being able to measure CRF expediently during a typical office visit health assessment or a population-based study would be valuable, it is a considerable challenge to adequately measure CRF in office medical settings or large-scale research investigations. Indeed, the increasing constraints of time, resources, and potential risk associated with assessing CRF by the "gold standard" method of graded exercise testing (GXT) make it all but impossible. Alternatives to a GXT are typically submaximal field tests. Examples of these include the Rockport 1-mile walk test [7] for estimating CRF, the Long Distance Corridor Walk (LDCW) [8,9], and the UKK Walk Test [10]. Although these field tests provide reliable estimates of peak oxygen consumption, they still carry some burden of time, resources, and risk. Because CRF is an important indicator of the ability of older adults to carry out essential activities of daily living [11] and potentially an important precursor to functional limitation and disability [8,12], identifying a relatively simple, low-cost, and low-risk measure of CRF could be of considerable benefit for use in clinical and research settings (e.g., for guiding exercise prescriptions).

Several research groups have developed equations that use variables associated with CRF such as age, sex, and physical activity level to approximate CRF in the absence of fitness testing [13-15]. Having a valid estimate of CRF could prove valuable in both healthcare and population-based research settings. Recently, Jurca et al. [16] have extended earlier attempts to develop equations to predict CRF using three very large samples (total N = 38,137). They expressed CRF in terms of metabolic equivalents (METs) and used several large data sets to establish the validity of a regression equation with the following components: age, sex, body mass index (BMI), resting heart rate (RHR), and self-reported physical activity (SRPA) level. Multiple correlation coefficients were similar across all data sets (*r *= 0.81, 0.77, 0.76) [16]. Moreover, the correlations between equation-estimated CRF in METs and METs measured by either maximal or submaximal graded exercise testing ranged between 0.74 and 0.80 for each sample. The original validation sample was characterized by participants who were primarily middle-aged (*M *age for men = 43.6 yrs; *M *age for women = 41.1 yrs) and relatively high fit (*M *measured METs for men = 11.72; *M *measured METs for women = 9.44).

The purpose of the present study was to test the construct validity of the regression equation developed by Jurca et al. [16] in a sample of older community-dwelling adults. The original equation was developed using primarily middle-age adults. However, given the rapidly increasing older adult population is it important to cross-validate the equation in this as well as other populations to maximize its applicability. To examine the construct validity, we performed a hierarchical regression analysis to determine the contribution of each of the individual equation components to measured METs. Additionally, we were interested in the extent to which the equation-estimated CRF value correlated with CRF measured by GXT, and how this relationship compared with the relationship between GXT-measured CRF and CRF estimated by a common field test. Finally, because CRF is often associated with cardiovascular conditions [17], we examined the association between self-reported conditions and each method of assessing CRF.

## Methods

### Participants

Participants were community-dwelling adults recruited from an ongoing study of cardiovascular fitness and brain structure and function. Recruitment took place via local media outlets, including television and print media advertisements. In order to participate in the present study, individuals had to meet the following inclusion criteria: aged 60 to 80 years, have no contraindications to submaximal or maximal exercise testing, and have no medical conditions exacerbated by physical activity participation. Following initial contact by telephone, participants completed a pre-screening interview to determine whether they met inclusion criteria and consented to have their physician contacted for approval to participate in exercise testing. Participants were excluded from participation if they did not meet the above criteria or their physician refused to provide approval for testing participation.

A total of 294 individuals were initially screened to participate in a study of aging and cognitive function. A total of 106 participants were excluded from the original sample due to the presence of medical conditions that may have been exacerbated by physical activity (e.g., knee/hip injuries, serious cardiovascular conditions) or circumstances that interfered with cognitive testing by magnetic resonance imaging (e.g., claustrophobia, metal objects in body). Of those who met the initial inclusion criteria, 16 did not complete the GXT (5 no longer interested, 3 had family problems, 7 could not get approval from their physicians, and 1 could not complete the GXT due to high blood pressure). Thus, 172 individuals completed the GXT and all measures necessary for the equation-predicted CRF. Nineteen of these participants did not attend or finish the Rockport test for a variety of reasons related to scheduling difficulties, inability to complete the test, or injuries unrelated to the GXT.

### Measures

#### Demographics

A brief questionnaire assessed basic demographic information including sex, age, education, income, marital status, and occupational status.

#### Maximal Graded Exercise Testing (GXT)

A physician-supervised GXT utilizing a modified Balke protocol [18,19] was used to assess peak oxygen consumption and obtain an objective measure of CRF. Participants walked at a self-selected brisk pace on a treadmill. The incline was increased every two minutes until the participant terminated the test volitionally or the physician stopped the test due to medical concerns. Expired gases were continually sampled and averaged over 30-second intervals throughout the test. A participant's VO_2max _was the highest value achieved when at least two of the following three criteria were met: a) a plateau of VO_2 _values, defined as an increase of <1.0 ml/kg despite an increase in power output, b) achieved age predicted maximal heart rate (220-age), and c) respiratory exchange ratio greater than 1.1. The highest MET value recorded by the metabolic measurement system during the GXT was used in subsequent analyses.

#### Submaximal Exercise Testing

The Rockport 1-mile walk protocol [7] was used as a submaximal estimate of CRF. This field test was conducted by trained staff with an ACLS certified nurse in attendance. Participants walked in groups on an enclosed, synthetic track, and were instructed to complete the 1-mile walk as quickly as possible without running. Cardiorespiratory fitness was estimated using the following standard Rockport 1-mile walk equations: a) Estimated VO_2 _(female) = 154.899 - (0.0947*2.2046*weight) - (0.3709*age) - (3.9744*walk time) - (0.1847*exercise heart rate); b) Estimated VO_2 _(male) = 116.579 - (0.0585*2.2046*weight) - (0.3885*age) - (2.7961*walk time) - (0.1109*exercise heart rate). All values were converted to METs for subsequent analyses.

#### Predicted Cardiorespiratory Fitness

Predicted CRF was calculated utilizing the original validation regression equation proposed by Jurca and colleagues: Estimated MET Value = Sex (2.77) - Age (0.10) - Body Mass Index (0.17) - Resting Heart Rate (0.03) + Self-reported Physical Activity + 18.07.

#### Self-Reported Physical Activity Index (SRPA)

SRPA was determined from a single exercise history question, as recommended by Jurca et al. [16]. Participants were asked to choose one of five activity categories that best described their usual pattern of daily physical activity, including activities related to home and family care, transportation, occupation, exercise and wellness, and leisure or recreation from the following: a) Level 1: inactive or little activity other than usual daily activities (Value = 0); b) Level 2: Regularly (≥ 5 d/wk) participate in physical activities requiring low levels of exertion that result in slight increases in breathing and heart rate for at least 10 minutes at a time (Value = 1); c) Level 3: Participate in aerobic exercises such as brisk walking, jogging or running, cycling, swimming, or vigorous sports at a comfortable pace or other activities requiring similar levels of exertion for 20 to 60 minutes per week (Value = 2); d) Level 4: Participate in aerobic exercises such as brisk walking, jogging or running at a comfortable pace, or other activities requiring similar levels of exertion for 1 to 3 hours per week (Value = 3); or e) Level 5: Participate in aerobic exercises such as brisk walking, jogging or running at a comfortable pace, or other activities requiring similar levels of exertion for over 3 hours per week (Value = 4).

#### Height and Weight

Height and weight were measured utilizing a Seca electronic scale and stadiometer (Model 763 1321139) at the GXT appointment prior to the start of the test. Participants were measured wearing light clothing and no shoes. BMI was calculated using the standard formula of weight (kg)/[height (m)]^2^.

#### Resting Heart Rate

RHR was collected at the GXT appointment prior to the start of the test utilizing a supine 12-lead EKG tracing. Participants rested quietly for approximately ten minutes before RHR was measured. Heart rate was recorded for ten seconds and the value utilized was the average of all R-R intervals within this duration.

#### Cardiovascular Conditions

A personal medical history was used to assess cardiovascular risk factors. Participants were asked to indicate whether or not they had experienced any of 13 conditions associated with an increased risk for cardiovascular disease (e.g., high blood pressure, shortness of breath, pain associated with poor circulation) by answering "yes" or "no". Yes responses were coded as a "1" and no responses were coded as "0". A cardiovascular conditions score was developed by summing all 13 items.

### Procedures

All procedures were approved by a university Institutional Review Board. An initial telephone screening call established criteria for study entry and collected medical history information. After receiving medical clearance from their personal physicians, participants signed an informed consent document and completed the demographic questionnaire at an orientation session prior to any testing. At the first testing session, all participants completed a physician-supervised GXT. Participants completed the Rockport 1-mile walk test at a second appointment, scheduled within 3 to 4 weeks of the GXT. RHR, BMI, and SRPA were determined at the GXT appointment.

### Data Analysis

Testing the construct validity of the CRF equation was conducted in several stages. First, we examined the descriptive characteristics of the sample, including their SRPA levels, in relation to the original validation samples. We conducted a correlation analysis in which we examined the associations between the equation-estimated CRF MET value and each component of the prediction equation. Next, we conducted a hierarchical regression analysis to determine the independent contribution of each of the CRF model components to overall MET obtained during the GXT. Subsequently, we examined the correlations between the MET values attained from the CRF equation and MET values from the GXT and sub-maximal exercise testing (i.e., Rockport test). We also conducted a repeated measures ANOVA to compare the mean MET levels obtained from the CRF equation, the GXT, and the Rockport test. In addition, we further examined the degree of agreement between the equation method and the GXT method by constructing a Bland-Altman plot [20]. Finally, we correlated the MET values from each of the three methods with the number of self-reported cardiovascular conditions.

## Results

### Participant Characteristics

Table 1 documents the demographic characteristics of the sample. The mean age of the sample was 66.7 years (S.D. = 5.7; range 58- 81 years). Participants were primarily female (63.4%) and married (57.6%). The majority of the sample was retired/working part-time (70.9%), White (89.5%), well educated (54.6% with at least a college degree), and with an annual household income greater than $40,000 (58.6%). Table 1 also includes the mean fitness levels determined by each of the three methods and participants' self-reported physical activity levels and cardiovascular conditions. For the GXT, two participants had a positive exercise test and the remainder terminated the GXT volitionally (i.e., the supervising physician did not intervene), and 79% of participants peaked (i.e., met at least two of the three criteria). For the Rockport test, time to complete one mile ranged from 11:30 to 26:05 minutes, with five participants being unable to complete due to fatigue or musculoskeletal pain. We detail the descriptive characteristics of key GXT and Rockport variables in Table 2. Overall, comparison of the present sample with the original validation sample reported by Jurca and colleagues [16] suggests our sample to be markedly older (66.7 yrs vs. 39.5 - 45.9 yrs), more overweight (BMI = 28.8 vs. 22.8 - 26.3), and less fit (6.1 METs vs. 9.03 - 12.61 METs).

**Table 1:** Descriptive statistics for study sample

Variable	Mean (standard deviation)/Frequency (%)
Demographics	
Age	66.73 (5.7)
Sex	
Male	63 (36.6%)
Female	109 (63.4%)
Body Mass Index (kg/m^2^)	28.8 (4.4)
Marital Status	
Married	99 (57.6%)
Significant Other	3 (1.7%)
Single	11 (6.4%)
Divorced/Separated	34 (19.8%)
Widowed	25 (14.4%)
Race	
Asian	6 (3.5%)
African American	12 (7.0%)
White	154 (89.5%)
Ethnicity	
Hispanic/Latino	3 (1.7%)
Non-Hispanic/Latino	169 (98.3%)
Education	
<College	78 (45.4%)
≥ College	94 (54.6%)
Income	
<$40,000	67 (41.5%)
≥ $40,001	95 (58.6%)
Fitness (METS)	
CRF predicted by GXT	6.17 (1.4)
CRF predicted by Rockport testing	6.12 (1.7)
CRF predicted by equation	6.12 (2.32)
Self-Reported Physical Activity Level	
1	55 (32%)
2	52 (30.2%)
3	22 (12.8%)
4	35 (20.3%)
5	8 (4.7%)
Cardiovascular Risk Factors	
None	27 (15.7%)
1-3	118 (68.7%)
4-6	25 (14.6%)
≥ 7	2 (1.2%)

**Table 2:** Descriptive statistics for key GXT and Rockport variables

Variable	Mean (SD)
GXT Variables	
VO2 (L/min)	1.73 (.53)
VO2 (mL/kg/min)	21.58 (4.90)
Maximum heart rate (beats/min)	156.78 (19.47)
Maximum respiratory exchange ratio (RER)	1.11 (.07)
Rockport variables	
Walk time (minutes)	17.05 (2.12)
Exercise heart rate (beats/min)	117.36 (16.17)
Body weight (kg)	79.82 (14.68)

### Correlations between CRF, Prediction Equation Components, and Other Measures of CRF

Table 3 shows the relationships between the equation-predicted CRF MET value and each of the equation components. The correlation between sex and METs (*r *= 0.67, *p *< .001) is considerably higher in the present sample than in the original validation samples (*r*s = 0.32 - 0.48). Correlations between METs and other equation components were generally lower and overall most closely resembled the 1990 Allied Dunbar National Fitness Survey sample [21] used in the Jurca et al. [16] study. To determine the independent contributions of each of the equation components to predicted MET values, we conducted a hierarchical multiple regression analysis. The overall equation was significant, *F *(3, 166) = 38.26, *p *= .0001, *r *= 0.73, *R*^2 ^= 0.54 (SEE = 0.97). The overall *R *is similar to all three of the samples used in the original validation study (*R*s = 0.81, 0.77, 0.76). Relative to individual component contributions, participant sex, age, and BMI were the strongest individual contributors to variance in METs. The SRPA level was also significantly associated with MET level. Resting heart rate, however, was not associated with MET level (see Table 4). These contributions are similar in magnitude to those reported in the Jurca et al. study, although resting heart rate was significantly associated with MET level in the original paper. All regression coefficients reported herein were standardized.

**Table 3:** Correlations (*p*) between equation components and equation predicted CRF

Equation Component	Equation Predicted CRF in METs
Physical Activity Level	.366 (.0001)
Age	-.220 (.004)
BMI	-.366 (.0001)
Sex	.670 (.0001)
Resting Heart Rate	-.247 (.001)

**Table 4:** Results of regression analysis

Equation Component	Standardized Beta	t	**Sig**.
Sex	.55	10.13	.000
Age	-.45	-8.39	.000
BMI	-.33	-6.04	.000
SRPA Level	.18	3.24	.001
RHR	-.05	-.86	.391

**Figure 1:**
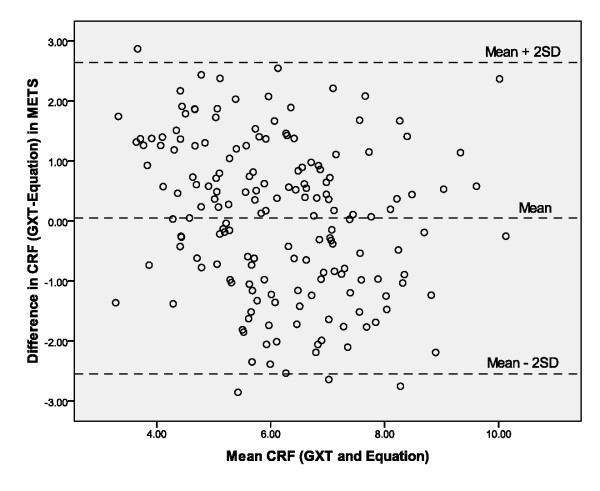
**Bland-Altman plot**.

Participants' CRF in METs, as predicted by the regression equation, was significantly correlated with their highest MET value attained by maximal graded exercise testing (*r *= 0.66, *p *< .001) and by the submaximal Rockport one-mile walk field test (*r *= 0.67, *p *< .001). The correlation between participants' CRF as predicted by the Rockport one-mile walk test and their GXT-measured CRF was also significant (*r *= 0.68, *p *< .001). When comparing errors of prediction between GXT-measured CRF and CRF estimated by the equation and the Rockport test, the error range was significantly smaller for the equation (-2.87 - 2.85) than for the Rockport test (-7.95 - 4.40). We then compared the agreement between CRF estimated from the predictive equation and CRF measured by graded exercise testing by plotting them using the Bland-Altman method [20]. As can be seen in Figure 1, this approach indicated that only 2.9% of the points were beyond ± 2 sds suggesting that the relationship is not driven by outlying values. The repeated measures ANOVA comparing the three measures of CRF was nonsignificant [F = .175 (1, 152), p = .84]. Follow-up analyses further revealed no significant differences between maximal MET levels determined by the GXT (*M *= 6.21, *SD *= 1.34, 95% CI = 5.99-6.42), the submaximal exercise test (*M *= 6.12, *SD *= 2.32, 95% CI = 5.75-6.50), or the equation (*M *= 6.19, *SD *= 1.67, 95% CI = 5.92-6.46).

### Correlations between MET Values and Cardiovascular Conditions

Finally, we correlated the three MET values with aggregated cardiovascular disease conditions in an effort to provide further evidence for the validity of the CRF equation (see Table 5). All three indices were significantly and inversely associated with reporting more cardiovascular conditions (*p *< .05). As can be seen, MET values calculated from maximal exercise testing were more strongly correlated with condition reporting than MET values from submaximal testing or the prediction equation. Comparison of these correlations was conducted using Fisher's Z-transformation which revealed no significant differences between each pair of correlations (*zs = 0.38-1.78, p > .05*).

**Table 5:** Correlations between CRF measures and cardiovascular conditions

CRF Measures	Number of Cardiovascular Risk Factors
Rockport Predicted CRF	-.177*
Equation Predicted CRF	-.218*
GXT Predicted CRF	-.361**

** p <.001; * p < .05

## Discussion

Cardiorespiratory fitness testing is costly, time-consuming, and, in the case of older adults, can necessitate some risk. These factors are prohibitive to routine clinical assessments of CRF and preclude use in larger population samples, in spite of the importance of CRF to numerous health outcomes [1]. The present study was designed to validate an equation developed by Jurca et al. [16] that combines a simple self-report physical activity index with age, sex, BMI, and RHR to estimate CRF in MET values [16] in a sample of community dwelling older adults. Initial development of this measure was conducted in several very large samples of community dwelling adults.

Our findings show the prediction equation yielded similar estimates of CRF as those reported in the original validation study and offer cautiously optimistic support for the use of this measure to estimate CRF in older adults. The correlation between equation-estimated CRF and CRF measured by GXT, the "gold standard," was relatively strong (*r *= 0.66) and almost identical to the correlation between CRF measured by graded exercise testing and CRF estimated from submaximal field testing. This latter finding alone suggests that assessment of CRF using this non-exercise method approximates the accuracy of using an established field test such as the Rockport one-mile walk to estimate fitness in older adults. It should be noted that the correlation between Rockport-estimated CRF and GXT-measured CRF in this sample (*r *= 0.68) is slightly lower than the correlation reported in the original validation paper for adults aged 60-69 (*r *= 0.74) [7]. In addition, other field-based measures such as the 400 meter walk of the LDCW correlate higher with measured CRF [8]. However, unlike the index validated herein, such measures still require participants to engage in exercise, and may not be practical for use with certain populations. Our findings deviated from those of Jurca et al. slightly in that resting heart rate was not a significant predictor of CRF in the present study. However, it must be noted that the regression coefficient for resting heart rate in the original validation paper was very small, suggesting that its statistical significance may have been driven by the large sample size.

We further tested the construct validity of the non-exercise CRF measure by examining the relationship of the MET values obtained from this measure and the maximal and submaximal exercise tests with self-reported cardiovascular conditions. As CRF is a reflection of efficient cardiovascular functioning, one would expect individuals with high CRF to have fewer cardiovascular conditions, and vice versa. All three measures of fitness were significantly associated with this measure, although the correlations with fitness measured by GXT were the strongest. This latter finding is to be expected, given that the GXT measures CRF most precisely. Although weaker, the correlations of cardiovascular conditions with estimated fitness obtained from the regression equation and submaximal methods were not significantly different from the association with the gold standard measure. More importantly, the correlation with the equation-predicted measure was almost identical to the correlation with the non-maximal measure, again suggesting that the non-exercise estimation of fitness is at least as strongly associated with cardiovascular conditions as a measure that requires significant resources in terms of medical supervision, time, cost, and exercise participation. If these findings can be replicated in other samples and when using other field and submaximal tests, this equation could be a very useful substitution to exercise testing, as it would significantly minimize the required resources.

The appeal of a non-exercise estimate of CRF is straightforward. The equation utilizes information routinely collected in a research laboratory, clinic, or physicians' practice to easily obtain an estimate of CRF and provide some initial indication of health status. For practitioners, the equation could be used as part of a fitness evaluation to provide more accurate and appropriate exercise recommendations that reflect initial fitness levels. For scientists conducting population-based research, such information would be particularly useful in examining associations between CRF and other important physical and mental health outcomes such as cognitive function, disability, and well-being. Although such potential outcomes are promising, continued validation evidence is necessary, particularly with older adults, other populations, and in larger samples. Moreover, it is of further interest to determine whether the equation-estimated CRF is predictive of change in fitness over time, either as a function of self-initiated behavior change or intervention participation. Finally, future studies should work towards determining a criterion estimated CRF level at which an individual is at greater risk for related health outcomes (CHD, mortality, etc.).

Our study is not without limitations. Our sample was composed largely of relatively healthy, white, female, well-educated, and economically advantaged community-dwelling older adults. However, the parent study from which the sample was drawn specifically recruited sedentary older adults and therefore the sample would appear to be representative of the older population. Replication in other adult populations is obviously warranted. Additionally, the equation does not consider other factors which may influence fitness levels such as genetic influences, prescription medications, or possible disease states. Although medications or chronic disease conditions could add to errors of prediction, the fact that participants were not disease- or medication-free also enhances the generalizability of our results to other community-dwelling older adult samples. One must also consider that Jurca et al. [16] used both maximal and submaximal exercise testing data to develop the original equation, and this likely introduced additional error to its predictive value. Finally, the self-reported physical activity level element, which is weighted heavily in the equation, is a subjective rating and, therefore, is open to the participant's interpretation. However, that self-reported physical activity makes a considerable contribution to the prediction of CRF adds further support to the validity of this aspect of the equation.

When performing a pre-exercise evaluation for at-risk populations or conducting research that necessitates precise measurement of CRF, it is recognized that there is no acceptable substitute for graded maximal exercise testing. There are several issues associated with using prediction equations, including determination of which is the "best" or most appropriate equation for a given population, potential lack of sensitivity to detect changes in CRF, and error associated with self-reported information. However, if a reasonable estimate of CRF is sufficient, and precise accuracy is not the primary concern, then the benefits of a non-exercise regression model such as that proposed by Jurca et al. [16] may be considerable.

## Conclusions

This research provides preliminary evidence to support the use of a low-cost, low-risk, and relatively expedient way of estimating cardiorespiratory fitness in relatively healthy older adults [16]. This estimate of CRF does not involve any exercise testing, provides similar estimates to a popular field test estimate of CRF, and could be of utility in both clinical and research settings.

## Competing interests

The authors declare that they have no competing interests.

## Authors' contributions

***EM: ***developed study concept and design, performed analysis and interpretation of data, involved in preparation of manuscript. ***ELM, SW, TW, and AS: ***involved in the acquisition of subjects and/or data, manuscript preparation. ***AK***: involved in manuscript preparationand study concept and design. All authors have read and approved the final manuscript.

## Pre-publication history

The pre-publication history for this paper can be accessed here:

http://www.biomedcentral.com/1471-2458/10/59/prepub
